# Investigation of Structural and Antibacterial Properties of WS_2_-Doped ZnO Nanoparticles

**DOI:** 10.1021/acsomega.3c09041

**Published:** 2024-01-05

**Authors:** Sercan Beytür, Sebnem Essiz, Bengü Özuğur Uysal

**Affiliations:** Faculty of Engineering and Natural Sciences, Kadir Has University, Cibali, Fatih, Istanbul 34083, Turkey

## Abstract

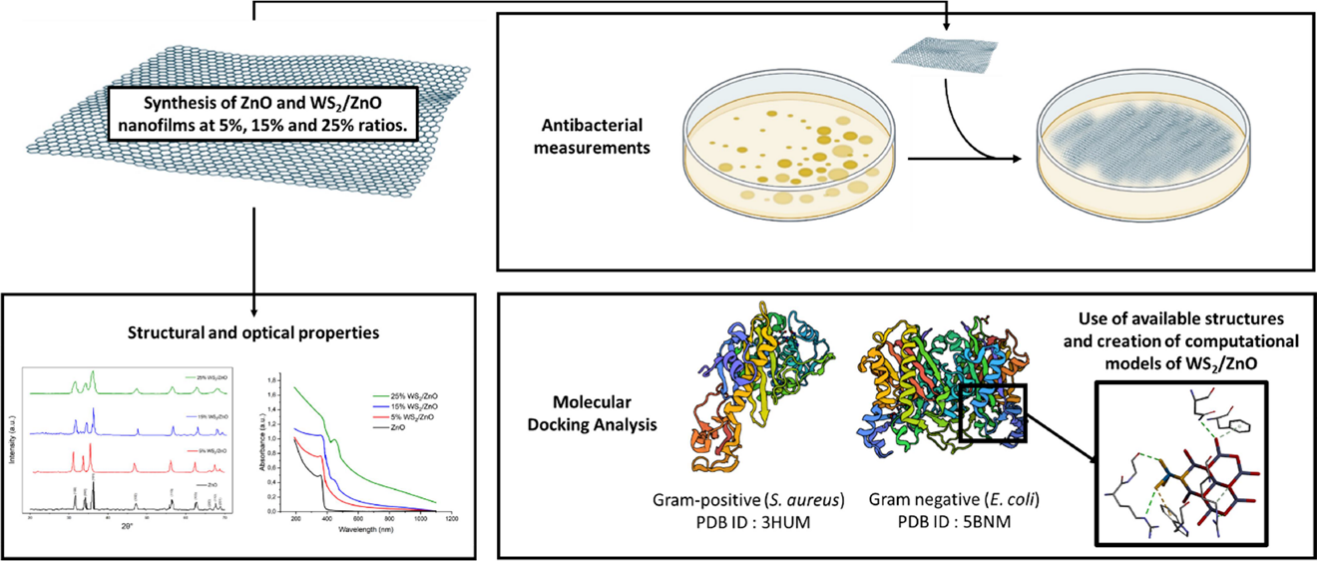

ZnO nanoparticles, well-known for their structural, optical, and antibacterial properties, are widely applied in diverse fields. The doping of different materials to ZnO, such as metals or metal oxides, is known to ameliorate its properties. Here, nanofilms composed of ZnO doped with WS_2_ at 5, 15, and 25% ratios are synthesized, and their properties are investigated. Supported by molecular docking analyses, the enhancement of the bactericidal properties after the addition of WS_2_ at different ratios is highlighted and supported by the inhibitory interaction of residues playing a crucial role in the bacterial survival through the targeting of proteins of interest.

## 1 Introduction

Metal oxides nanoparticles (NPs) became attractive for a large variety of applications including catalysis, sensors, electronic materials, environmental remediation, or biomedical applications for diagnoses and therapeutics, e.g., in tissue therapy, immunotherapy, dentistry, regenerative medicine, wound healing, and biosensing platforms.^1−4^ With their tiny size and broad surface-to-volume ratio, encouraging them to interact directly with microbial membranes and not merely because metal ions are released via solutions, the antibacterial effect of metal oxide NPs has also been demonstrated.^5^ This antibacterial application of metal oxide NPs has recently been reviewed by Naseem and Durrani.^6^ To support the study of the antibacterial response of different metal oxide composites, molecular docking studies gained importance through the investigation of protein–ligand interactions with the aim of highlighting amino acids of the targeted protein’s active site and supporting the hypothesis of a consequent inhibitory effect leading to a bactericidal observation.^7−9^

Because of their physical and chemical properties, zinc oxide (ZnO) NPs have gained considerable interest: high electrochemical stability, super oxidative capability, and low toxicity.^10,11^ They crystallize in two main forms: hexagonal wurtzite, which is most stable under ambient conditions, has excellent thermal and mechanical stability, and is thus more common than the cubic zincblende form. ZnO possesses a wide bandgap in the hexagonal wurtzite structure (around 3.37 eV), and the exciton binding energy (around 60 meV) is large at room temperature and became a widely used semiconducting material as a UV-sensitive material.^12−14^ ZnO is also the first and most used material for heterogeneous photocatalysis among other metal oxides and has been used as an antibacterial agent for a variety of biomedical purposes, such as bioimaging, drug delivery, gene delivery, and biosensors.^15,16^ Antibacterial properties of ZnO on Gram-positive and Gram-negative bacteria have also been investigated in another example of the study.^17^

Besides the potential of metal oxide NPs, specific physical and chemical properties of two-dimensional (2D) materials also made them of interest as materials used in various applications.^18,19^ Different methods such as mechanical exfoliation, Scotch tape and gel-assisted mechanical exfoliation, chemical vapor deposition, and femtosecond laser irradiation permit production of 2D materials, which have higher strength compared to 3D materials, as well as a higher ratio of surface area to volume, increasing their rate of reactions. Superconductivity in 2D materials has also become one of the attractive research areas in recent years due to their excellent properties, flexibility, and ability to be stacked into layers in vacuum atmospheres.^20^ The diversity in the 2D material properties also plays an important role in fabricating heterostructures with 2D superconducting contacts. Due to their properties, the most investigated 2D materials are transition metal dichalcogenides (TMDs), and mechanically exfoliated TMDs (such as MoS_2_, WS_2_, NbSe_2_, and so on) have been broadly studied.^21^

Tungsten is the heaviest transition metal, and its association with sulfur atoms, forming tungsten disulfide (WS_2_) in the family of common TMDs, became an emerging 2D nanomaterial.^22−24^ Layered WS_2_ is composed of a strong particle covalent bond (S–W) and a weak van der Waals force with an interlayer spacing of 0.7 nm.^25−29^ WS_2_ has a P63/mmc hexagonal crystal layer and at room temperature, the stability of the WS_2_ structure is due to the strong S–W–S covalent bonds.^30−38^ It has a low bandgap that can change from an indirect bandgap (1.3 eV) to a direct and higher bandgap (∼2 eV) when the thickness approaches the monolayer.^39^ The band gap energy can be controlled by the number of layers, which confers to WS_2_ nanomaterial's great application potential in the field of optics.^40,41^ The antibacterial activities of WS_2_ nanosheets against two representative bacterial strains, Gram-negative *Escherichia coli* and Gram-positive *Staphylococcus aureus*, were evaluated by colony-forming unit (CFU) studies.^42^ The enhanced biological behavior and antibacterial property of WS_2_ nanosheets modified mesoporous bioactive glass nanospheres for bone tissue engineering.^43^

2D semiconductor materials have an excellent absorption capacity in the visible light region, which significantly enhances the absorption capacity of ZnO, and the addition of WS_2_ appeared to improve ZnO-containing nanohybrid properties. Different synthesis methods and usages of ZnO/WS_2_ nanohybrids have been established. Environmental remediation applications have been studied through the study of WS_2_/ZnO photocatalytic action with the aim of removing organic pollutants.^44,45^ The enhancement and development of UV photodetectors have also been explored,^46,47^ and a significant enhancement of photosensitivity in the short wavelength range has been shown.^48^ A study on the effect of WS_2_ nanosheet addition on the performance of the ZnO nanorod-based photodetector presented an enhancement of UV-detector performance sensitivity increasing from 129 to 334%.^46,49^ The WS_2_/ZnO composite has also been explored as an optical fiber taper and can induce normal dispersion mode-locking, indicating that the WS_2_/ZnO composite is suitable for generating high-powered mode-locked pulses.^50^ An antibacterial application has also been observed in graphene oxide/WS_2_/Mg-doped ZnO nanocomposites for solar-light catalysis.^51^ The WS_2_/ZnO nanohybrids exhibit considerably improved antibiofilm activity and inhibited the biofilm formation, with 1.5 times higher activity compared to pristine WS_2_ nanosheets, suggesting that the nanohybrid materials are more effective as novel antifungal materials.^52^

Through experimental measurements of microstructural and optical properties, in addition to antibacterial effect measurement and the support of a molecular docking study targeting representative strains of Gram-positive (*S. aureus*) and Gram-negative (*E. coli*) bacteria, the present study aims to spotlight the antibacterial effect of ZnO nanosheets after addition of WS_2_ at various ratios. It was expected to provide a promising new composite with superior structural, optical, conductive, and antibacterial features.

## 2 Materials and Methods

### 2.1 Structural and Crystallographic Properties of the Films

#### 2.1.1 Materials

A ZnO solution was prepared by dissolving zinc acetate dehydrate (ZnAc) in isopropanol. Dea (diethanolamine), which is a surface-active material, was used to accelerate solving. Water was added for hydrolysis reactions, and as a precursor solution of ZnAc/isopropanol/Dea/water, a volume ratio of 0.4:4:0.1:0.2 was used. The solution was mixed using magnetic stirring for 1 h at 60 °C. Then, one-half of the obtained solution was deposited on Corning 2947 glass substrates by spin-coating deposition (1000 rpm/30 s), using a spin coater, at room temperature (22 °C) and denoted as the ZnO film after the annealing.

The rest of the ZnO solution was used to prepare composite films, mixing with WS_2_ powder at various ZnO/WS_2_ ratios (5, 15, and 25%). All chemicals were provided by Sigma-Aldrich Co LLC. All are in liquid form, except ZnAc and WS_2_. To add only 4 mL into 40 mL of solvent, zinc acetate dihydrate was weighed as 6.96 g and added to the solution. Similarly, WS_2_ powder was weighed as 860, 2580, and 4310 mg and added to the solutions for 5, 15, and 25%, respectively. The final solutions were deposited on Corning 2947 glass substrates by spin-coating deposition (1000 rpm/30 s) using a spin coater at room temperature (22 °C). After coating, ZnO and ZnO/WS_2_ films were immediately placed in a microprocessor-controlled (CWF 1100) furnace heated at 500 °C. The films were taken out of the furnace and left at room temperature at the end of 1 h. Finally, all coatings and heat treatment processes were repeated two times to get three-layered films.

#### 2.1.2 X-ray Diffraction

The microstructural properties were analyzed using an X-ray diffraction (XRD, Philips PW-1800) diffractometer with CuKα radiation at λ = 1.5406 Å. XRD is a technique that reveals structural information, such as the crystal structure, crystallite size, chemical composition, and strain. It can be used to analyze thin films and powders.

Since the X-rays are waves of electromagnetic radiation, some of these waves cancel one another out in most directions, and some of them are strengthening other waves in a few specific directions. This relationship is determined by Bragg’s law:
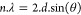
1Here, *d* is the spacing between crystal planes (interplanar spacing), θ is the incident angle, *n* is the integer, and λ is the wavelength of the X-rays.^53^ To calculate the diameter of the nanocrystals, Debye–Scherrer’s equation is used:^54^
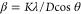
2where β is the full width at half-maxima, *K* is the constant of X-ray source (0.89), and *D* is the diameter of nanocrystals.

The thicknesses of the films were measured by using a stylus profilometer (Veeco, Dektak 150). To take thickness measurements, we first attached tape to an area of approximately 0.5 cm from one edge of the substrate glass. Afterward, the films were coated with a spin-coating method by dropping the solution on them, and then the tape was peeled off from the edge of the substrate glass. In this way, a step was created in the edge of the glass. Thickness measurement of the films was carried out with the help of step formation, starting from this edge with a profilometer. The same measurement was repeated for each sample.

The surface morphology of the films were determined using scanning electron microscopy (SEM)

### 2.2 Optical Properties of the Films

To study optical properties, absorption spectra were recorded using a PerkinElmer Lambda 900 ultraviolet–visible (UV–vis) spectrophotometer at room temperature. UV–vis spectroscopy is an analytical method for a large class of organic and inorganic compounds. Nondestructive, simple, and inexpensive, UV–vis spectrophotometer determines the absorbance of the material or the transmittance (*T*) of light passing through a medium. It is used to understand materials’ optical features, in a solution or a solid phase.^55^ This device tracks the excited atoms by looking at their wavelengths and absorbance energies. The UV–vis spectrophotometer detects the absorbance of the film between the range of the 200–1100 nm spectrum.^56^ From absorbance, transmittance can then be determined as follows:
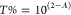
3

Urbach tail energy, *E*_u_, can be evaluated from the slope of the linear fit of the absorbance curve plotted against the wavelength of the incident light.^57^ The curve should have a section of a straight line, and if extended to the *x* axis, the *x* intercept of this line gives the wavelength for which the absorbance is null. This wavelength is used for dividing the constant photon energy (*E* = *hv* = 1239.3 eV):
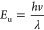
4

The following formula is needed for converting absorbance intensity into the absorption coefficient, α:
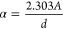
5where *A* is the absorbance value and *d* is the thickness of the film.

A Tauc plot is one method of determining the optical bandgap in semiconductors.^58,59^
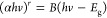
6where *hv* is the energy of a photon, *B* is a constant, α is the absorption coefficient, *E*_g_ is the bandgap energy, and *r* gives the information about the transition. For the ZnO composite film that has a direct transition, the Tauc equation is revised as follows:
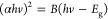
7

The square of the product of the absorption coefficient and photon energy is plotted versus the photon energy for the direct transition semiconductors. The curve should have a section of a straight line. The extrapolation of the curve with respect to the energy axis points the special energy out. This energy is called the optical bandgap energy of the material.

### 2.3 Antibacterial Activity

Antibacterial measurements can be performed using a method based on comparing the colony numbers of the bacteria by contacting the film surface with them. For this, the surfaces of the films must be smooth, and the films to be measured must be 1 cm thick including the substrate glass and a square shape of 5 × 5 cm^2^. Six samples of a Corning glass covered with WS_2_ (5, 15, and 25%)-doped ZnO thin films were prepared for the experiment, three samples to test bacteria and three to control groups. The antibacterial effect of various concentration of WS_2_ (5, 15, and 25%) in the presence of ZnO have been used for studying the survival of Gram-positive (*S. aureus*) and Gram-negative (*E. coli*) bacteria. Each of these samples was analyzed by an ISO 22196. The antibacterial activity of the films was evaluated by CFU counting. After incubation, the colonies were counted. CFU per milliliter was calculated for each sample at different time intervals (0–120 min) by using the following formula:
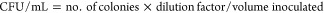
8

### 2.4 Molecular Docking Study

#### 2.4.1 Biological Targets

The 3D structures of β-lactamase from Gram-positive (*S. aureus*) (PDB ID: 3HUM) and a β-ketoacyl–acyl carrier protein synthase III (FabH) from Gram-negative (*E. coli*) bacteria (PDB ID: 5BNM) are obtained from the PDB databank.^60−63^ β-Lactamase and FabH are proven to be essential for bacterial survival and growth with their role in biosynthesis of the cell wall and fatty acids, respectively. Thus, their inhibitors have been reported as potent antibiotics, and the identification of inhibitors against these targets may contribute to the discovery of new antibiotics.

#### 2.4.2 Ligands

The Materials Project online platform was used to match the structure. The best coherent structure for ZnO was mp-2133 (https://materialsproject.org/materials/mp-2133/), and the structure matching with the WS_2_ commercial powder used in the experiments was mp-224 (https://materialsproject.org/materials/mp-224/). A WS_2_/ZnO structure model has been created combining both computational crystal structures on Chemcraft software.^64^ In addition to single WS_2_/ZnO, supercell structures containing a total of eight ZnO or WS_2_/ZnO structural model at 12.5 and 25% different ratios (containing 1 WS_2_/7ZnO and 2 WS_2_/6ZnO, respectively) were made using VESTA software.^65^ This supercell calculation would be used to understand the behavior of different WS_2_/ZnO ratios on the binding site interactions. The PM6 calculation method of partial charges has been computed on MOPAC2016.^66^

#### 2.4.3 Molecular Docking

Molecular docking was performed using AutoDock4 through AutoDockTools (1.5.6 version).^67^ This was performed for each ligand as a single molecule structure and as a supercell structure composed of eight molecules. The locations of 3HUM and 5BNM active sites are obtained through the coordinates of their associated reference ligands, which are removed from the binding pocket to make room for docking our ligands of interest. A gridbox size of 40 × 40 × 40 and 60 × 60 × 60 Å have been defined, respectively, for the single molecule structure and the supercell. The number of genetic algorithm runs has been defined as 20. From the 20 best docked conformations, the best scored one is retained to visualize amino acids involved in the ligand–protein interaction in Discovery Studio Visualizer (BIOVIA, Dassault Systèmes).

## 3 Results and Discussion

### 3.1 Structural and Optical Properties

#### 3.1.1 Structural Properties

**Figure 1:**
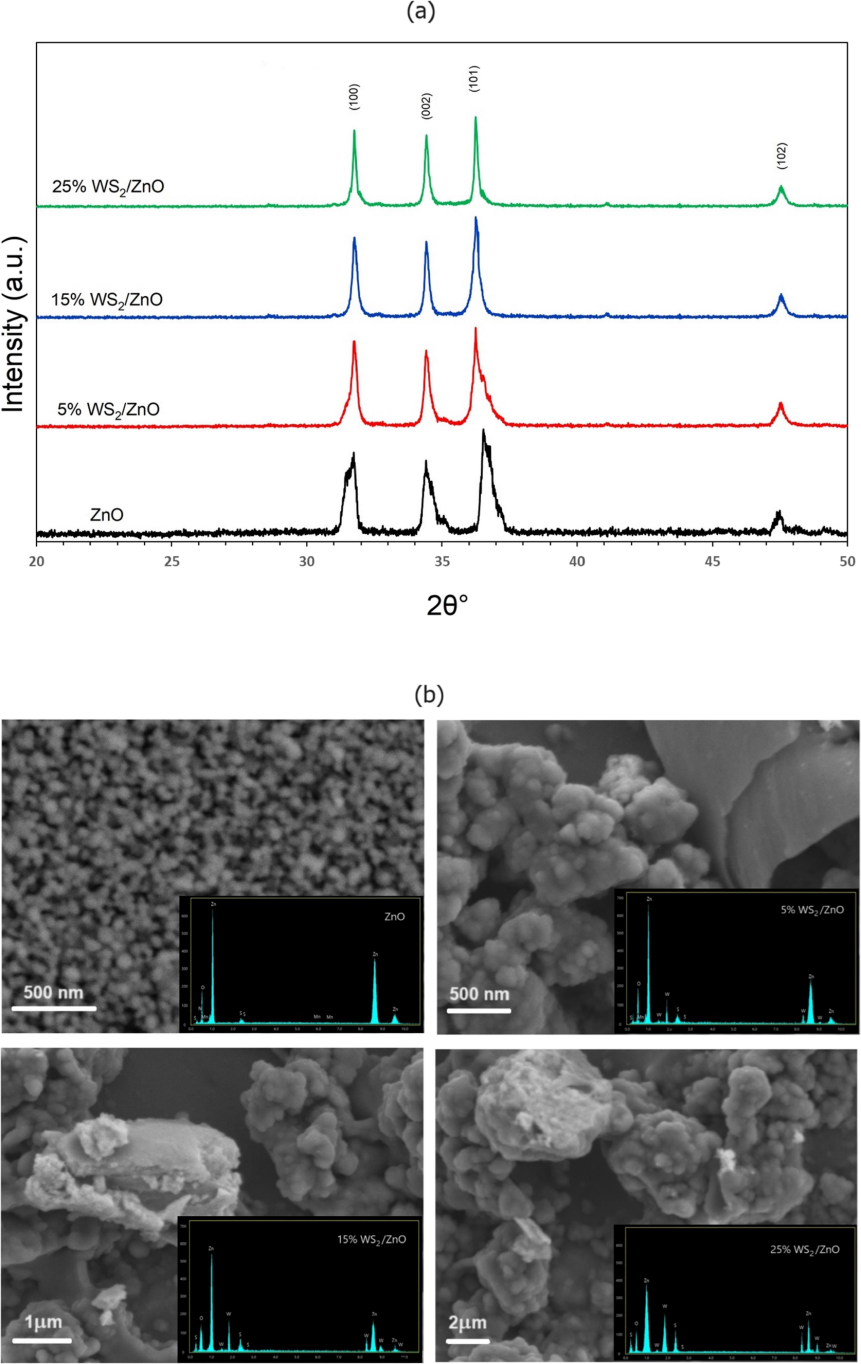
(a) XRD patterns of ZnO and ZnO doped with WS_2_ at 5% (in red), 15% (in blue), and 25% (in green) ratios. (b) SEM images of ZnO and WS_2_/ZnO films. Insets: EDS elemental analysis of the films.

The XRD pattern of ZnO and ZnO/WS_2_ in Figure 1a shows the sharp peaks corresponding to a zinc oxide indexed as a hexagonal wurtzite (ICDD card no. 36-1451) and confirms the polycrystalline nature of the synthesized ZnO. Furthermore, due to a limitation of conventional XRD instruments to detect too thin structure components, an absence of peak showing the WS_2_ or other related phases is to notice. There are four diffraction peaks at 2θ of 31.76, 34.42, 36.24, and 47.54° corresponding, respectively, to (100), (002), (101), and (102) crystal planes for ZnO nanoparticles for the range of 2θ = 20.00–50.00°. The addition of WS_2_ does not significantly affect the position of these diffraction peaks. The peak at the (101) crystal plane has the highest intensity. Followed by (100) and (002) crystal planes, these three peaks have the highest intensities compared to other diffraction peaks for ZnO nanoparticles also after the addition of WS_2_ at different ratios.

Average crystallite sizes of ZnO were then calculated from (100), (002), and (101) crystal planes. Microstructural properties of ZnO did not present significant changes after the addition of WS_2_. From these data and the Scherrer equation, the calculated diameter of ZnO nanocrystals is around 17.17 nm, and the addition of WS_2_ to ZnO nanocrystals augments this diameter from 32.61 to 58.31 nm (Table 1).

**Table 1:** Structural Properties of ZnO and ZnO Doped with WS_2_ at 5% (in Red), 15% (in Blue), and 25% (in Green) Ratios

	average crystal size (nm)	thickness (nm)	band energy (eV)	Urbach tail energy (eV)
ZnO	17.17 ± 1.24	522	3.29	3.29
5%WS_2_/ZnO	32.61 ± 2.81	734	3.17	3.17
15%WS_2_/ZnO	41.26 ± 3.12	752	2.85	2.96
25%WS_2_/ZnO	58.31 ± 4.24	801	2.56	2.62

Elemental analysis of the WS_2_/ZnO films have been examined by EDS, which was performed during the SEM measurements; the result is presented in Figure 1b. It is evident that the WS_2_/ZnO films consist of WS_2_ sheets decorated with many nanoparticles. The EDS image (inset in Figure 1b) of the ZnO film reveals the presence of Zn and O elements with little impurity signals as expected for the ZnO film. In the insets of Figure 1b, it is observed that the WS_2_/ZnO films are mostly composed of W, S, Zn, and O elements. The results discussed above prove the successful process of creating WS_2_/ZnO films.

ZnO nanoparticles and micrometer-sized sheets of WS_2_ are clearly visible in the SEM images (Figure 1b). When the surface morphology of the undoped ZnO film in the upper left picture of Figure 1b is examined, the presence of nanoparticles with sizes varying between 13 and 32 nm is observed. In all other SEM images, WS_2_ sheets and the ZnO nanoparticles surrounding them can be seen agglomerated from one region to another. Elemental analysis conducted in different regions confirmed the observations. However, agglomerated nanoparticles and sheets did not provide a reliable particle distribution. On the other hand, when XRD results are compared with surface morphology and elemental analysis, WS_2_ does not contribute to the crystal behavior and the crystallite size calculated using the Scherrer relation according to XRD measurements may correspond to the size of ZnO nanoparticles, and WS_2_-doped films have larger crystallite sizes as the amount of doping increases. It is thought that it may be caused by WS_2_/ZnO agglomeration in the presence of WS_2_ sheets. In a previous research, the WS_2_/ZnO composite, hybrid,^45^ or heterojunction^46−48^ structure has been examined in terms of surface morphologies and it was determined that it had very different behaviors (structurally nanoparticle and sheet). In addition, by taking advantage of this difference, it was discovered that they have interfacial charge transfers,^43^ carrier regulation characteristics,^45^ and fast optical response.^49−52^

### 3.2 Optical Properties

A considerable increase in nanofilm thickness has been measured (+171 nm) after the addition of WS_2_ to ZnO nanoparticles at a percentage of 5%, while multiplying this percentage of WS_2_ by three or five is adding 50 and 92 nm to the thickness of WS_2_/ZnO nanofilms. This difference in composition and thickness impacts the optical properties (Table 1).

**Figure 2:**
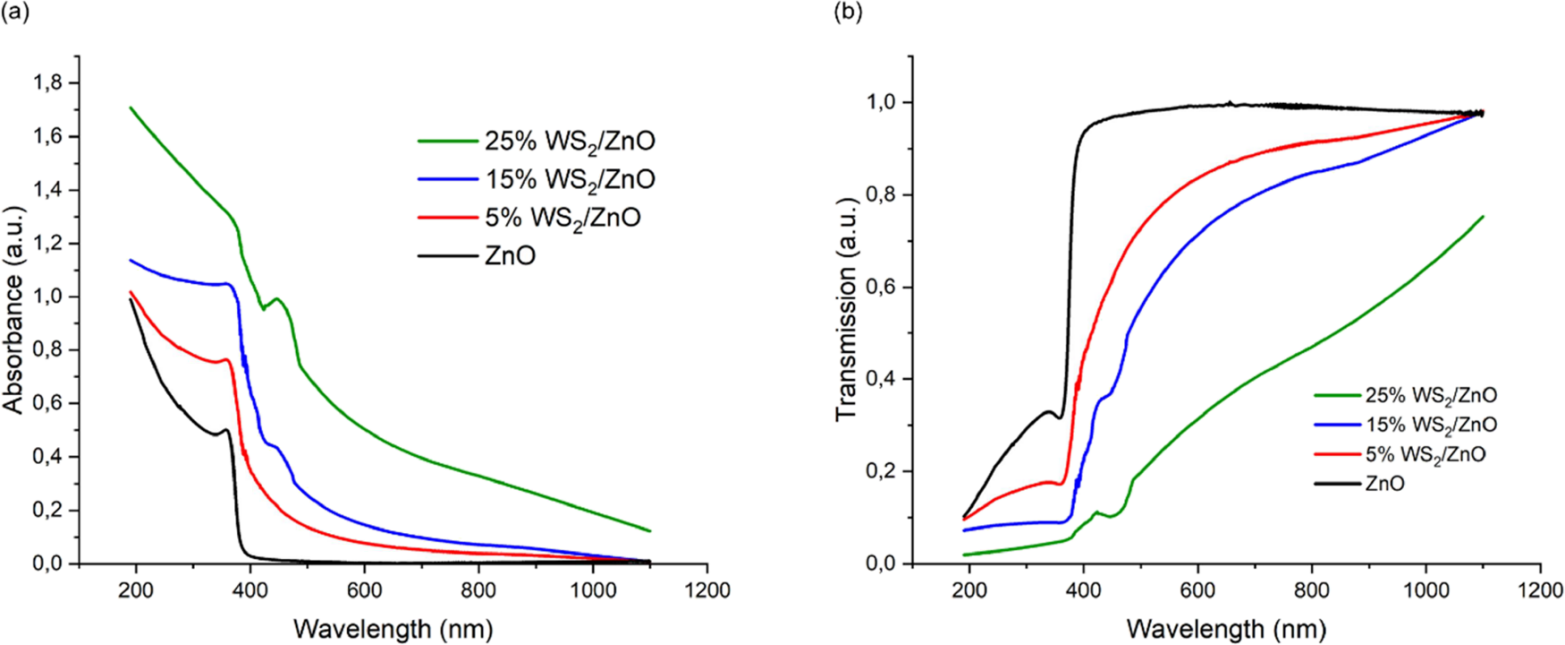
Absorption (a) and transmission (b) spectrum of ZnO and ZnO doped with WS_2_ at 5% (in red), 15% (in blue), and 25% (in green) ratios.

The absorbance spectrum of ZnO and after addition of WS_2_ shows high absorption (Figure 2a) and low transmission in the UV-light region (Figure 2b). However, the addition of WS_2_ results in an enlargement of the absorption and transmittance areas into the visible light region (400–700 nm) and beyond, in the infrared light region, when containing 25% of WS_2_. With 15 and 25% of WS_2_, in the blue light region (around 430 to 490 nm), an increase in absorption and a drop in transmittance are noticeable. This is an effect of WS_2_ since it is a low bandgap semiconductor that absorbs visible light.

**Figure 3:**
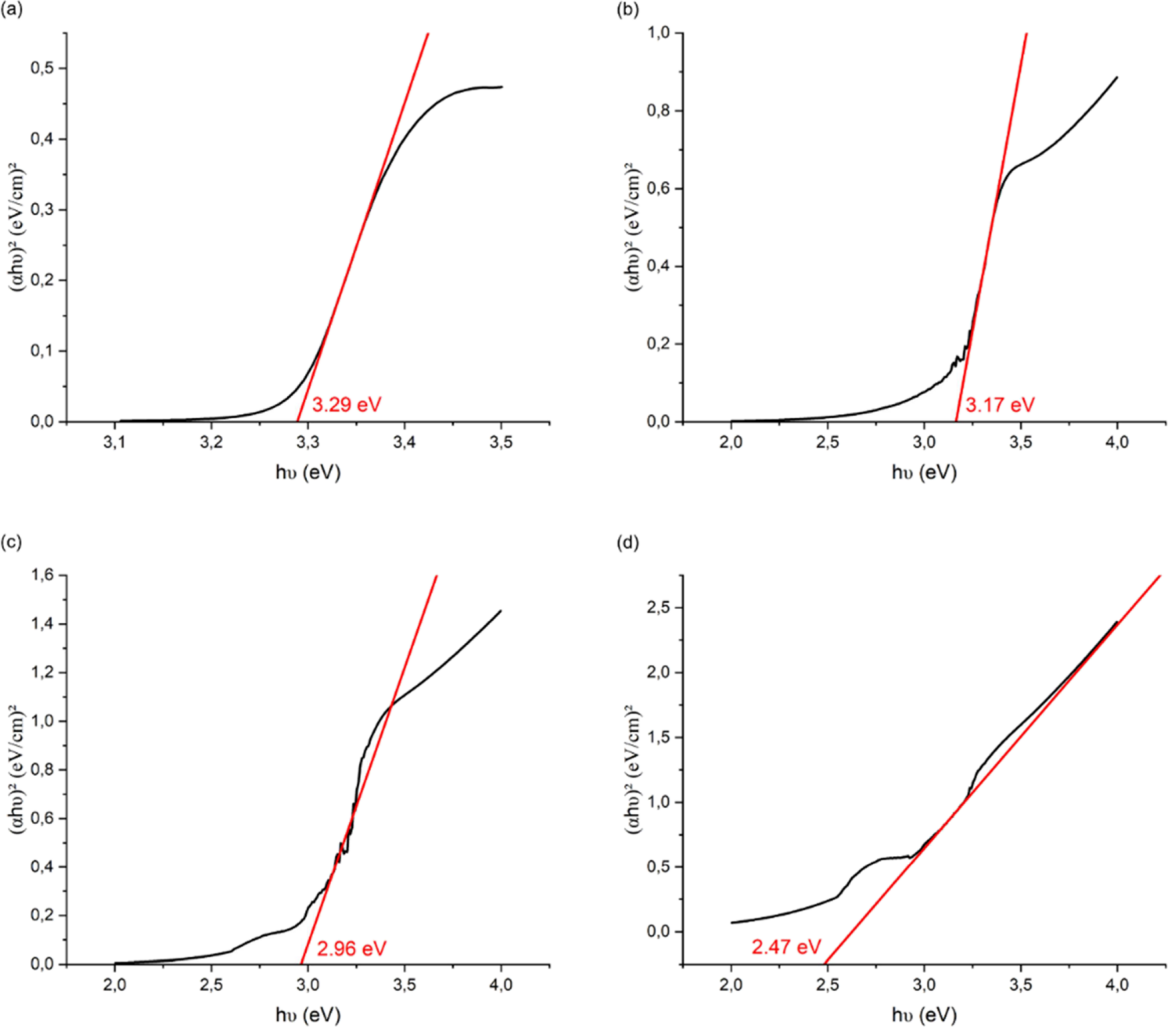
Tauc plot and bandgap energy determination of ZnO (a) and ZnO doped with WS_2_ at 5% (b), 15% (c), and 25% (d). The bandgap energy values are in red.

The optical bandgap energy of ZnO nanostructures is known to range from 3.10 to 3.37 eV and has been calculated to be ∼3.29 eV (Figure 3).^68−71^ The doping of ZnO with WS_2_ lowers the bandgap energy, and this drop is more or less important depending on the percentage of WS_2_ in ZnO/WS_2_ thin films. While the lowest percentage of WS_2_ is still having a bandgap energy at 3.17 eV and being able to absorb at the UV region, higher percentages of WS_2_ present a decrease in bandgap energy, around 2.85 and 2.56 eV. A larger bandgap means that at the end that light of a higher frequency and lower wavelength would be absorbed; thus, the observed decrease corresponds to a shift in an ability to absorb more in the visible light region. It also shows the increased influence of WS_2_, which is known to have, as a monolayer, a calculated bandgap at 2.3 eV and a band edge absorption located in the visible light region. This decrease in bandgap energy after the addition of WS_2_ is in line with other studies, which also leads to the hypothesis that an electronic interaction in the ZnO/WS_2_ nanohybrid may cause the observed bandgap reduction.^72,73^ Increasing the WS_2_ loading amount in the ZnO/WS_2_ nanohybrid, the S_2_– ions create oxygen vacancies in the ZnO structure.^74,75^

### 3.3 Antibacterial Effect of WS_2_/ZnO

#### 3.3.1 Measurement of the Antibacterial Effect

**Figure 4:**
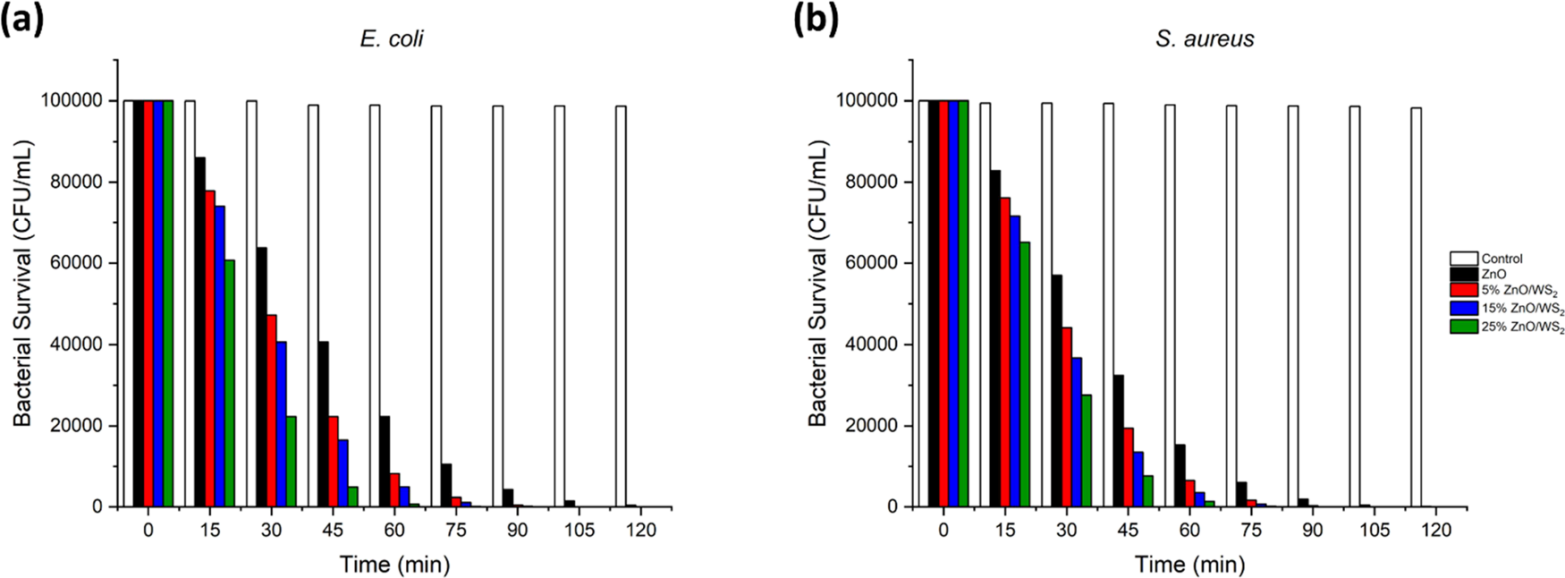
Bar graphs of *E. coli* (a) and *S. aureus* (b) survival (CFU/mL) after being in contact with the surface of ZnO (in black) and ZnO doped with WS_2_ at 5% (in red), 15% (in blue), and 25% (in green).

The survival of the representative species of Gram-negative bacteria, *E. coli*, and Gram-positive bacteria, *S. aureus*, has been measured and after the addition of ZnO, the concentration in both types of bacterial colonies decreased (Table 2 and Figure 4). The bacterial survival is more than halved after 45 min for both bacterial species (around 41,000 CFU/mL for *E. coli* and 32,000 CFU/mL for *S. aureus*) and becomes null after 120 min for *E. coli* and after 105 min for *S. aureus*. Consistent with the literature, the observed antibacterial properties of ZnO nanoparticles, on both bacterial types, have been widely studied through different methods (e.g., disk diffusion, broth or agar dilution, and microtiter plate-based method).^15,76−78^ To go further, beyond the use of individual ZnO nanoparticles in current biomedical applications, combinations with other materials (metal oxide nanoparticles or metal doping, as well as biomaterials such as chitosan, silk sericin, gelatin, and others) are explored.^79^ A bacterial environment being important in these types of applications, the bactericidal properties of ZnO combined with other structures, such as graphene oxide, present a significantly lower normalized viability ratio of bacteria incubated with ZnO/graphene oxide than those incubated with individual ZnO.^80^

**Table 2:** Number of CFU of *E. coli* and *S. aureus* after Being in Contact with the Surface of ZnO- and WS_2_-Doped ZnO Nanofilms at 5, 15, and 25% Ratios at Different Timepoints

	bacterial survival (CFU/mL)									
	*E. coli*					*S. aureus*				
time (min)	control	ZnO	ZnO/WS_2_ (5%)	ZnO/WS_2_ (15%)	ZnO/WS_2_ (25%)	control	ZnO	ZnO/WS_2_ (5%)	ZnO/WS_2_ (15%)	ZnO/WS_2_ (25%)
0	100,000	100,000	100,000	100,000	100,000	100,000	100,000	100,000	100,000	100,000
15	99,957	86,025	77,835	74,036	60,653	99,466	82,857	76,085	71,608	65,098
30	99,955	63,717	47,191	40,611	22,313	99,454	56,933	44,078	36,742	27,600
45	98,961	40,611	22,268	16,484	4878	99,348	32,420	19,423	13,488	7597
60	98,960	22,268	8163	4933	663	99,025	15,290	6494	3522	1331
75	98,765	10,494	2306	1065	54	98,806	5960	1627	628	116
90	98,761	4240	479	138	3	98,733	1904	280	46	0
105	98,760	1454	46	2	0	98,659	479	3	0	0
120	98,707	406	3	1	0	98,229	72	0	0	0

The doping of ZnO with WS_2_ at different ratios enhances the bactericidal effect. Indeed, with 5% WS_2_/ZnO, the concentration in bacterial colony is halved after 30 min (around 47,000 CFU/mL for *E. coli* and 44,000 CFU/mL for *S. aureus*). Despite a slight enhancement of the antibacterial effect, the increase from 5 to 15% WS_2_/ZnO does not present a major difference and the bacterial survival is null after 90 min for both species. However, the increase to 25% WS_2_/ZnO presents a striking enhancement of the bactericidal effect after 15 min: with a value around 61,000 CFU/mL, a difference around 17,000 CFU/mL with 5% WS_2_/ZnO and around 13,000 CFU/mL with 15% WS_2_/ZnO is to notice. After 30 min, only a quarter of the population survived (approximately 22 000 CFU/mL) and the survival is almost null after an hour. The contact of WS_2_ nanosheets with a bacterium cell membrane is known to cause serious damage to its integrity, leading to the cell death. However, it has been shown that the reactive oxygen species generated by WS_2_ nanosheets are modest regardless of the WS_2_ concentration.^42^ The improvement of the bactericidal activity due to the implication of WS_2_ is consistent with the literature, such as the suggested improvement of graphene oxide/WS_2_/Mg-doped ZnO nanocomposites' antibacterial activity.^51^

#### 3.3.2 Molecular Docking Study

To understand the structural properties of antibacterial activities observed in the experimental measurements, molecular docking analyses were performed. Docking a single molecule of ZnO or WS_2_ on both biological targets, free energy of binding ranges between −1.42 and −1.68 kcal/mol (Table 3). The free energy of binding is improved when a WS_2_ is combined with ZnO as a single molecule docked to the proteins: −2.18 kcal/mol with 3HUM and −2.07 kcal/mol with 5BNM. A similar comment can be made about the supercell structures. The ZnO supercell presents a free energy of binding of −3.37 and −3.34 kcal/mol for 3HUM and 5BNM, respectively. However, this estimated free energy of binding is enhanced in the presence of WS_2_ at 25%, the highest concentration used in antibacterial activity measurements (−5.45 and −4.39 kcal/mol after docking on 3HUM and 5BNM, respectively). An intermediate of 12.5% ratios used in antibacterial activity measurements has been proposed, namely, a single WS_2_ plus seven ZnO, and presents an improved binding score: −4.95 and −4.25 kcal/mol after docking on 3HUM and 5BNM, respectively. These enhancements suggest an improved inhibition of the biological targets due to the addition of WS_2_ and the identification of the amino acids interacting with our ligands can highlight this inhibitory effect.

**Table 3:** Free Energy of Binding (kcal/mol)

individual molecule						supercell (eight molecules)					
3HUM			5BNM			3HUM			5BNM		
ZnO	WS_2_	WS_2_/ZnO	ZnO	WS_2_	WS_2_/ZnO	8ZnO	1WS_2_/7ZnO (12.5%)	2WS_2_/6ZnO (25%)	8ZnO	1WS_2_/7ZnO (12.5%)	2WS_2_/7ZnO (25%)
–1.68	–1.62	–2.18	–1.55	–1.42	–2.07	–3.37	–4.95	–5.45	–3.34	–4.25	–4.39

Active sites of penicillin-binding proteins, such as our first biological target, are characterized by a set of conserved motifs, including a Ser-X-X-Lys (SXXK) tetrad containing the serine nucleophile, the Ser-X-Asn (SXN) and the Lys-Thr(Ser)-Gly (KTG) triads.^61^ In the previous study of two antibiotics, hydrolyzed ampicillin and cefotaxime, binding poses are emphasizing Ser75, Ser139, and Lys259 catalytic residues.^81^ In Table 4, interactions of our metal ligands and binding site residues are summarized. The addition of WS_2_ to ZnO involves different amino acids by offering additional possibilities of interaction types, such as π–sulfur interaction due to sulfur atoms (Table 4).

**Table 4:** Amino Acids Interacting with the Docked Ligand and Interaction Type^a^

	3HUM						5BNM					
	individual molecule			supercell			individual molecule			supercell		
interaction type	ZnO	WS_2_/ZnO	WS_2_	ZnO	12.5%	25%	ZnO	WS_2_/ZnO	WS_2_	ZnO	12.5%	25%
hydrogen bonds		SER139 GLU297	SER139 THR260 ARG300	LYS249 GLU297 LYS298	SER75 GLU297	GLU297		HIS244 ASN274	HIS244 ASN274	TRP32 ARG36	ARG36 ARG151 GLY152 ASN247	THR35 ARG36
π–sulfur interaction			TYR268		PHE241	PHE241 TYR268 TYR291		HIS244	PHE157 HIS244		TRP32	TRP32
electrostatic interaction	GLU297			GLU297	GLU297		ASP150		PHE308			
carbon hydrogen bonds		SER262		LYS298	GLY261	GLY261	GLY152			ARG36	ARG36	
metal acceptor		ASN269			SER262							
π-donor hydrogen bonds						PHE241				PHE213	PHE213	

a Colors of the interaction type correspond to the colors of the dotted-line observed in Figures 5 and 6.

On 3HUM, the docking of single ZnO presents an electrostatic interaction (with GLU297), while WS_2_ forms hydrogen bonds (with SER139, THR260, and ARG300) and π–sulfur interactions with TYR268. The docking of an individual WS_2_/ZnO exhibits hydrogen bonds (with SER139 and GLU297), carbon–hydrogen bonds (with SER262), and a metal–acceptor interaction (with ASN269). The docking of the ZnO supercell structure also presents an electrostatic interaction with GLU297 as well as a hydrogen bond with this amino acid, LYS249 and LYS298. A carbon–hydrogen bond is also formed between the ZnO supercell structure and LYS298. The addition of WS_2_ to ZnO at a ratio of 12.5 or 25% is forming both hydrogen bonds with GLU297 and π–sulfur interaction with PHE241 besides other interactions.

On 5BNM, an electrostatic interaction with ASP150 and a carbon–hydrogen bond are observable after the docking of an individual ZnO. The formation of an electrostatic bond is also noticeable with the docking of a single WS_2_ besides hydrogen bonds with ASN274 and HIS244. A π–sulfur interaction is also built with HIS244 and with PHE308. Hydrogen bonds are seen with the same residues as with WS_2_ after the docking of a single WS_2_/ZnO. A π–sulfur interaction is also noticeable with HIS244 only. The ZnO supercell structure interacts with TRP32 and ARG36 through hydrogen bonds. ARG36 is also having hydrogen bonds with the supercell containing WS_2_ at 12.5 and 25% ratios besides other residues. ARG36 is also interacting via a carbon–hydrogen bond with ZnO and 12.5%WS_2_/ZnO. Moreover, a π-donor hydrogen bond is also formed with PHE213 with the previously mentioned ligands. Common to the supercell containing WS_2_, π–sulfur interaction with TRP32 is to notice.

The molecular docking of single WS_2_/ZnO (Figure 5a–c) presented interactions with SER262 (carbon–hydrogen bond or metal–acceptor interaction), SER139 (hydrogen bond), GLY261 (carbon–hydrogen bond), and GLU297 (electrostatic interaction), which are members of the active site and/or known to interact with inhibitory compounds (Table 4). In WS_2_-containing ZnO structures at ratios of 12.5% (Ser75, Phe241, Gly261, Ser262, and Glu297) and 25% (Phe241, Gly261, Ser262, Tyr268, Tyr291, and Glu297), similar interacting residues are also observed (Figure 6a–c).

**Figure 5:**
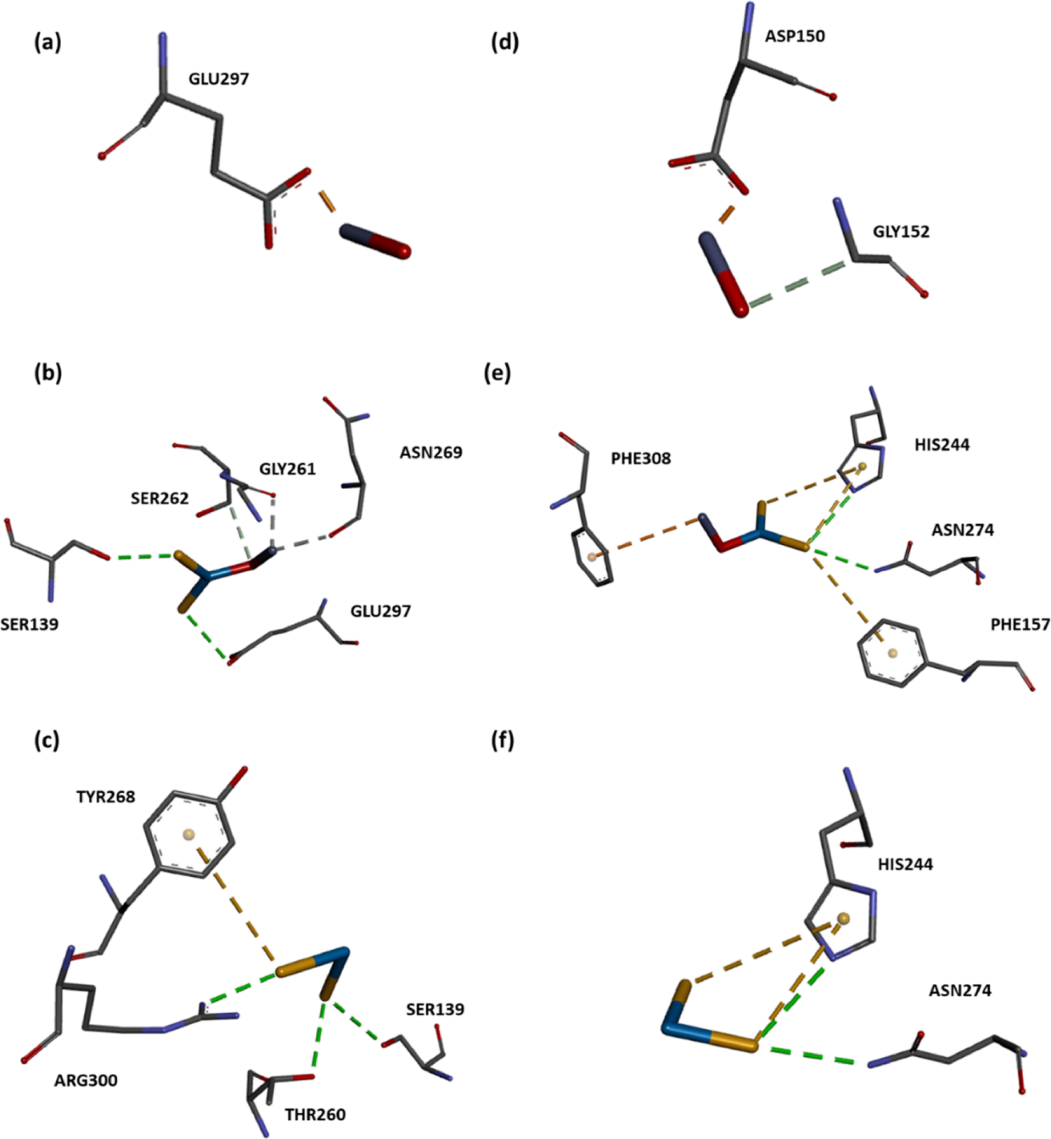
Molecular docking of single ZnO, WS_2_/ZnO, and WS_2_ on 3HUM (a–c) and 5BNM (d–f). Interactions are represented as dotted lines, and their types are listed in Table 4.

**Figure 6:**
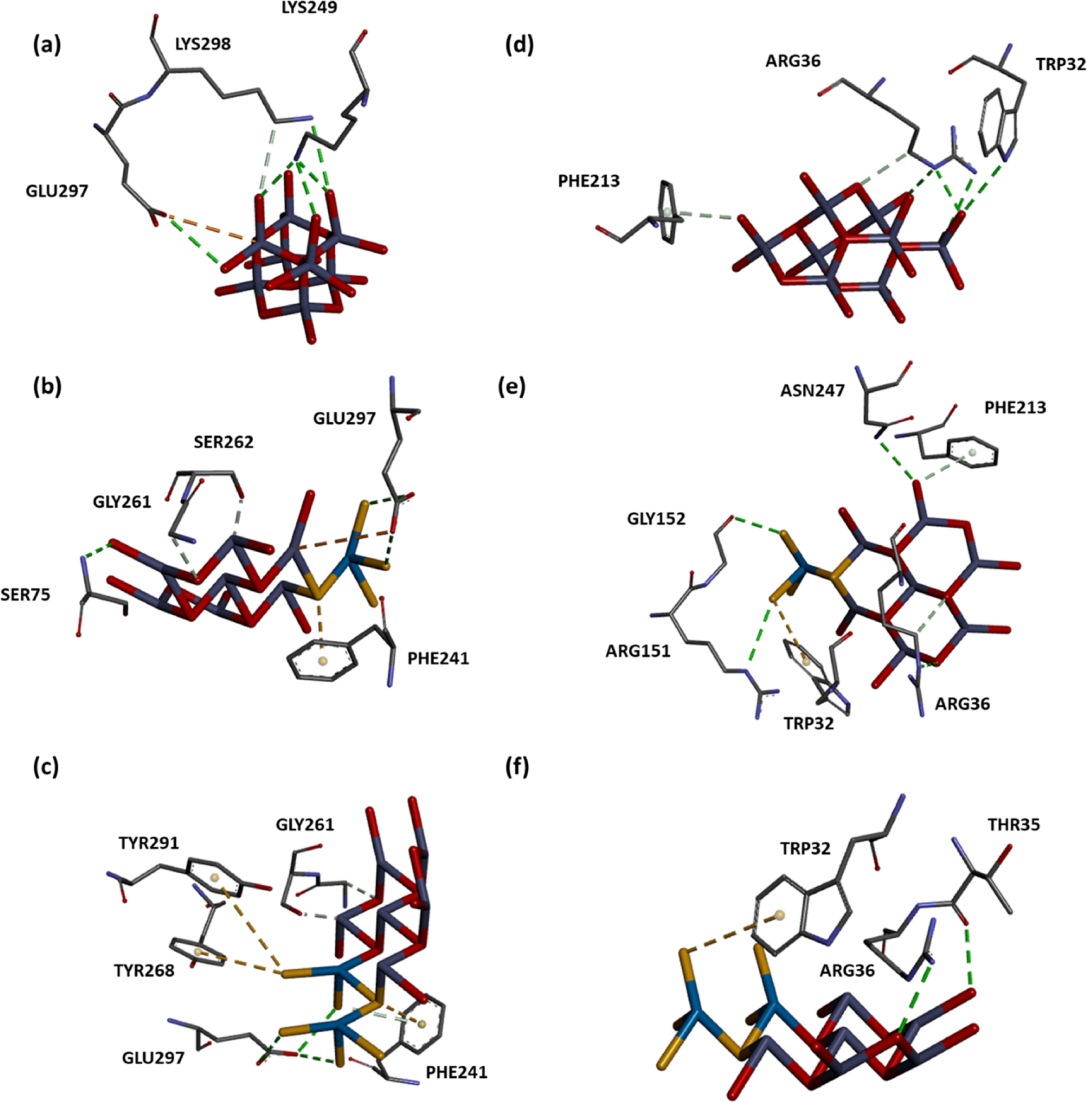
Molecular docking of supercell structures containing eight ZnO only or containing WS_2_ at 12.15 and 25% on 3HUM (a–c) and 5BNM (d–f). Interactions are represented as dotted lines, and their types are listed in Table 4.

The active site of FabH (5BNM) is composed of the Cys112, His244, and Asn274 catalytic triad for which a mechanism of a condensation reaction has been proposed.^82,83^ The FabH class of type II fatty acid synthesis condensing enzymes (FASII) possesses a Cys-His-Asn catalytic triad. The FabH class of enzymes also condenses acyl-CoA with malonyl-ACP to initiate FASII, vital for bacterial membrane biogenesis. This condensation reaction occurs after the acyl chain has been transferred to the active site cysteine and the subsequent binding of malonyl-ACP.^84^ In a catalytic triad, histidine is the most common base, which contributes to the deprotonation of the nucleophile. The importance of basic His244 in decarboxylation and condensation reactions has been demonstrated and is known to interact with the inhibitory compound.^85^ Molecular docking of single WS_2_/ZnO shows an interaction with residue members of the catalytic triad His244, Asn274 besides Phe157, and Phe308 (Figure 5d–f).

Besides the catalytic site of FabH, an active site tunnel entrance composed of Trp32, Arg36, Arg151, and Phe213 has also been defined and they have been highlighted in past studies as interacting with inhibitory compounds.^86,87^ In the present study, these residues are all interacting with our 12.5% WS_2_/ZnO ligand and Trp32 and Arg36 are also interacting with 25% WS_2_/ZnO, which suggests an inhibitory effect (Table 4 and Figure 6d–f). Thus, not only are binding site residues blocked but also the active site tunnel entrance is affected by the WS_2_/ZnO, namely the entrance of small molecules to the active site would be also affected.

**Figure 7:**
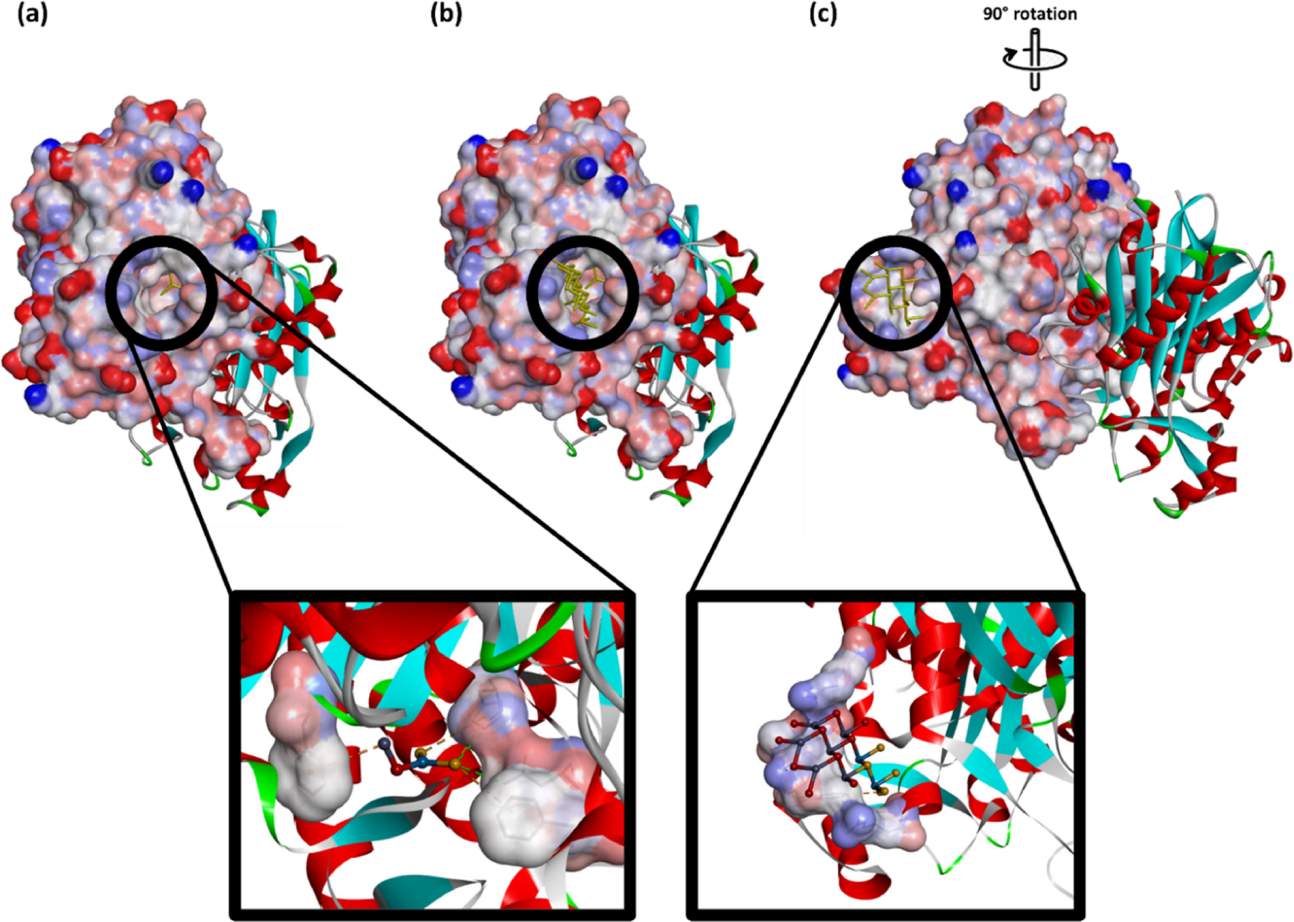
Localization of the individual WS_2_/ZnO (a), 25% WS_2_/ZnO supercell and individual WS_2_/ZnO (b), and the supercell only after rotation to the left (c) on FabH of *E. coli*(PDB ID: 5BNM). Chain A of the protein is represented as the surface and chain B as ribbon. Zoomed pictures represent the ligand as balls and sticks, the interacting amino acids as surface, and the protein as ribbon.

Moreover, for the proper functioning of FabH, a possible dimerization with an interface primarily formed by four loops of residues (84–86, 146–157, 185–217, and 305–307) have also been proposed.^88,89^ Residues that are important for substrate recognition include Trp32 and Arg151 (and Phe87 of the other monomer). Trp32 and Arg151 lie at the entrance to the active site. As a nanosheet, WS_2_/ZnO does not reach to the residues of the catalytic triad but it can block the active site tunnel entrance or disallow the dimerization and the proper function of the enzyme, leading to an antibacterial effect (Figure 7).

## 4 Conclusions

Films composed of ZnO NPs and ZnO NPs doped with WS_2_ at 5, 15, and 25% ratios have been synthesized, and microstructural properties of ZnO did not present significant changes after addition of WS_2_; its integrity is maintained. The increase in the average crystal size diameter and film thickness after the addition of the dopant affects the optical properties of the synthesized nanofilms.

Absorbance and transmittance at the UV-light spectra are enhanced, and the addition of WS_2_ results in an enlargement of the absorption and transmittance areas into the visible light region. This is consistent with the lowering of the bandgap energy while increasing the percentage of the dopant.

UV-light is known for its antimicrobial properties, and besides the optical properties, the enhancement of the bactericidal properties, after addition of WS_2_ at different ratios, is highlighted after being in contact with the synthesized nanofilms. This bactericidal activity is supported by molecular docking analyses, and the affinity is better after the addition of WS_2_ than with individual ZnO. This can be explained by the involvement of amino acids of interest in the functioning of the targeted bacterial proteins.
